# Unveiling a Unique Posterior Cloaca Variant: Expanding the Spectrum

**DOI:** 10.1055/a-2733-3072

**Published:** 2025-11-14

**Authors:** Tarlia Rasa Govender, Emanuele Trovalusci, Andre Theron, Chris Westgarth-Taylor, Giulia Brisighelli

**Affiliations:** 1 Department of Paediatric Surgery, Nelson Mandela Children's Hospital, Johannesburg, South Africa; 2 Department of Paediatric Surgery, Chris Hani Baragwanath Hospital, Johannesburg, Gauteng Province, South Africa; 3 Department of Paediatric Surgery, Charlotte Maxeke Johannesburg Academic Hospital, Johannesburg, Gauteng Province, South Africa; 4 Pediatric Colorectal and Urogenital Program, Baylor College of Medicine, Houston, Texas, United States

**Keywords:** posterior cloaca, urorectal malformation sequence, aphallia

## Abstract

A posterior cloacal variant is a congenital malformation where a urogenital sinus terminates anterior to a normally placed anus. These are rare malformations with highly variable anatomy. We report on three cases of a novel phenotype of posterior cloaca encountered at our institutions between October 2021 and November 2023. Three newborn girls were referred with ambiguous external genitalia and an anorectal malformation. In all cases, a midline sac, which is likely fused labioscrotal folds, replacing the clitoris was noted anterior to the perineal orifices. Two of the three patients demised as a result of renal failure. The third patient underwent reconstruction and is well. This posterior cloacal phenotype appears to be frequently associated with severe renal insufficiency. In survivors of the neonatal period, a good cosmetic outcome is achievable. Functional outcomes remain to be assessed.

## Introduction

**Fig. 1:**
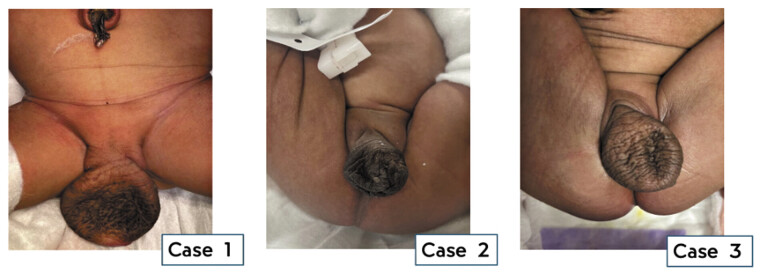
Midline fused labioscrotal folds forming an anterior sac devoid of corpora.

A posterior cloaca is defined as a urogenital sinus which diverges posteriorly and terminates in the anterior rectal wall. 1 When the urogenital sinus opens anterior to a normally placed anus, it is considered a posterior cloaca variant ( Fig. 1 ). 1 The description of this rare anorectal malformation is highly variable in terms of the anatomy of the external genitalia, number of perineal orifices, and aberrant Müllerian structures, with associated renal and cardiac defects. 1 We present three cases with similar unusual external genitalia, anorectal malformation, and renal insufficiency ( Table 1 ). Reconstruction in the surviving patient involved transperineal total urogenital mobilization and introitoplasty, sparing the anal canal.

**Table 1:** Summary of the perineal anatomy and associated anomalies of the patients

Case 1	Case 2	Case 3
- Fused labioscrotal folds - No corporal tissue - No palpable gonads **→ Two** perineal orifices	- Fused labioscrotal folds - No corporal tissue - No palpable gonads **→ Single** perineal orifice	- Fused labioscrotal folds - No corporal tissue - No palpable gonads **→ Two** perineal orifices
Hydrocolpos Two hemivaginas	Hydrocolpos Bicornuate uterus	Hydrocolpos
Bilateral hydronephrosis	Bilateral hydronephrosis	Solitary left kidney Accessory urethra
______	Distended bladder	Distended floppy bladder
Atrial septal defect	Patent ductus arteriosus	______
______	Deficient abdominal muscles	Deficient abdominal muscles

## Case 1

**Fig. 2:**
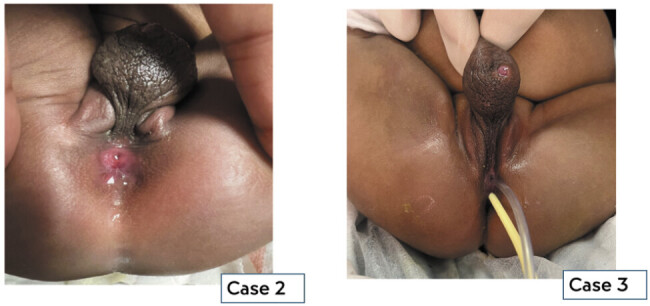
Perineal view demonstrating two orifices with the posterior orifice (anus) partially or fully within sphincters.

A term neonate born at 2,975 g was referred on day 3 of life. On arrival she was clinically stable and passing urine and stool via two closely situated perineal orifices, separated by a common wall. The posterior orifice was partially surrounded by sphincters. Anterior to these orifices was a large midline sac devoid of corpora and gonads, consistent with large fused labioscrotal folds ( Figs. 1 and 2 ). There was no catherizable channel within this structure. Urgent surgical intervention included a midline laparotomy, with a vaginostomy, drainage of bilateral hydrocolpos with division of vaginal septum, and a divided colostomy. On postoperative day 3, she required a relook laparotomy for abdominal wall dehiscence, during which a suprapubic catheter was placed for bladder decompression. Imaging of the urinary system demonstrated bilateral hydronephrosis with mild hydronephrosis on the right and moderate on the left. The urinary bladder was noted to be collapsed and there was no evidence of residual hydrocolpos. Despite these interventions, the patient succumbed to renal failure a few days later.

## Case 2

A term neonate with prenatal diagnoses of bilateral hydronephrosis, anhydramnios, and pulmonary hypoplasia was delivered at our center. The parents had declined second-trimester termination of pregnancy, and no antenatal chromosomal analysis was performed. At birth the child was found to have deficient abdominal wall musculature, bilateral clubfeet, ambiguous genitalia with a midline fluid-filled sac in place of a clitoris, and a single perineal orifice ( Fig. 1 ). A plain radiograph demonstrated a poorly developed sacrum. At laparotomy, hydrocolpos of a single vagina, a bicornate uterus, and a markedly distended bladder were documented. She underwent diversion with a divided colostomy, vaginostomy, and vesicostomy. Intra- and postoperatively, the patient had persistent severe metabolic acidosis and was anuric which led to a fatal outcome.

## Case 3

A term baby girl was referred at 3 weeks of age with ambiguous genitalia and an anorectal malformation. Antenatal ultrasound at 35 weeks had revealed hydronephrosis of a solitary left kidney and a single umbilical artery. Postnatal chromosomal analysis confirmed a 46 XX karyotype. Post-delivery she was well and not requiring any organ support. The child was feeding and passing stool and urine. On examination, she had a mildly distended abdomen and a palpable left kidney. Her perineal examination included a hyperpigmented anterior sac with rugae which was fluid filled and located between the labia majora. Posterior to the sac were two perineal openings: an anterior opening draining urine and a posterior, normally sited anus surrounded by well-developed sphincters (see Figs. 1 and 2 ). A plain radiograph demonstrated a normal sacrum.

She underwent examination under anesthesia and laparotomy. At laparotomy, she underwent a vaginostomy for hydrocolpos of a non-septated vagina, a vesicostomy for a distended bladder, and an end colostomy. A divided colostomy was deemed unnecessary, given the clearly patent and appropriately located anus. Postoperative recovery was uneventful. The diagnostic workup for associated congenital anomalies included a renal ultrasound, which demonstrated resolving hydronephrosis of her single left kidney and a structurally normal heart on echocardiogram.

**Fig. 3:**
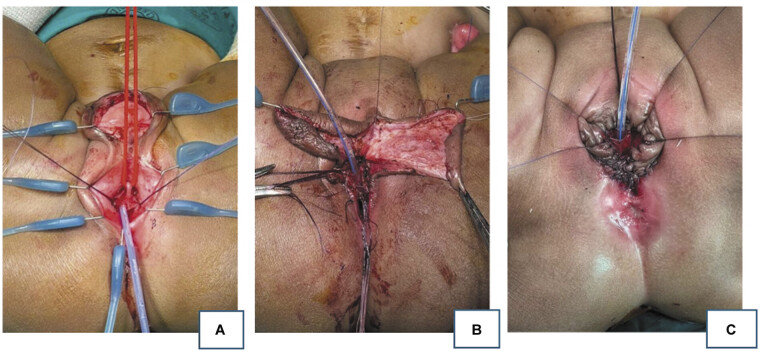
Case 3: Intraoperative photos. Left to right in supine position with anterior sagittal incision. ( **A** ) Urogenital sinus held by two sutures, Foley catheter in orthotopic urethra and red vessel loop around dorsal atretic accessory urethra. ( **B** ) Introitoplasty: Labioscrotal sac opened, and two halves fashioned into labia minora. ( **C** ) Final result with preserved native anus, perineal body created, and visible urethral and vaginal openings at perineum.

**Fig. 4:**
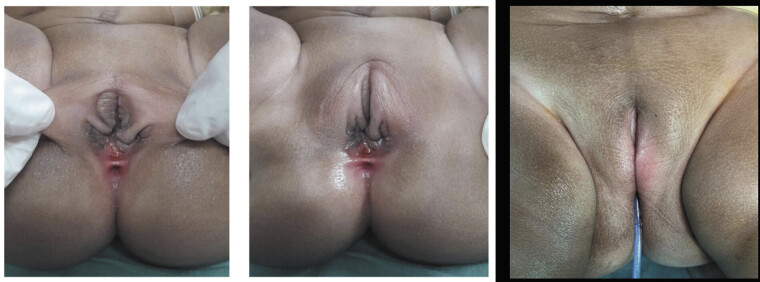
Case 3: 1 month post reconstruction with good cosmetic outcome.

At the age of 7 months, she underwent cystovaginoscopy and contrast studies for surgical planning. Our center offers two-dimensional fluoroscopy, which was used to plan the reconstruction. Pertinent findings included a thick pubic bone and an anus within sphincters that calibrated to a Hegar 14 with a normal dentate line. An accessory urethra was suspected due to urine drainage from the sac but was not initially identified. A transperineal total urogenital mobilization (TUM) was performed with the patient in supine position. Stay sutures were placed around the urogenital sinus and circumferential incision was made to begin mobilization. The incision was extended anteriorly though the midline of the sac. During mobilization of the urogenital sinus the orthotopic urethra was identified and catheterized. On opening the sac structure, accessory rudimentary urethra was seen. The proximal extent was unclear, and it was ligated and excised. Despite the presence of pubic symphysis hypertrophy, there was no need to resect any cartilage or bony elements to create space for the mobilization. Genitoplasty was achieved by midline division and folding of the labioscrotal sac around the TUM forming labia minora around the introitus. The anus was left untouched. The vaginostomy tube was removed. Urine was diverted via the vesicostomy and a transurethral catheter for 1 month postoperatively ( Fig. 3 ). The cosmetic outcome was satisfactory (see Fig. 4 ), and postoperative cystovaginoscopy confirmed a normal urethral length and one patent vaginal orifice. At 18 months old she underwent closure of her colostomy. Her continence outcomes are yet to be assessed as she is not yet toilet trained.

## Discussion and Conclusion

**Fig. 5:**
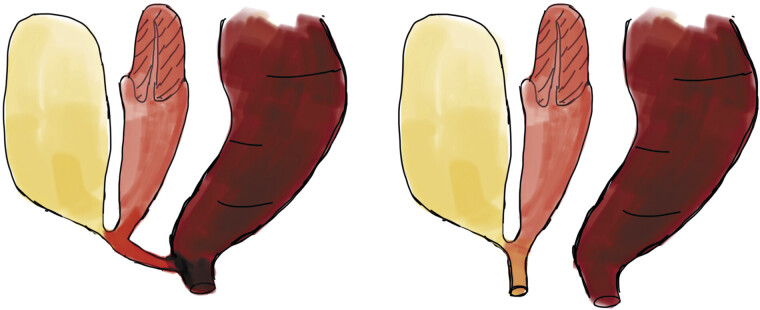
Schematic representation of posterior cloaca types.

In the original 1998 description of posterior cloaca, Peña and Kessler described both types of posterior cloaca, noting that the posterior cloaca variant (two orifices) may be more accurately classified as a variant of the urogenital sinus rather than a true cloacal malformation. 2 Fig. 5 However, because both types of posterior cloaca share features and associated abnormalities typical of cloaca patients, the authors recommended including them within the broader spectrum of cloacal malformations. 1 2 Furthermore, in 2010, Peña et al proposed that the posterior cloaca in females and aphallia in males may represent parts of the same spectrum, given the phenotypic similarities. 1 In both patient groups, there is typically a prominently developed pubic bone, a posteriorly displaced urogenital sinus, and a spectrum of hypoplastic orthotopic urethra, ranging from complete absence to varying degrees of urethral stenosis. These conditions have been collectively referred to by some authors as “partial uro-rectal septal defects.” 3

In cases with a single perineal orifice, the main differential diagnosis are: typical cloacal malformation, where the orifice opens at the level of the urethra or just above it; classic posterior cloaca, where the orifice opens in the position of a normally located anus; and penile agenesis, which can phenotypically resemble posterior cloaca but is usually distinguishable by the presence of palpable gonads within the scrotum. 4 When two perineal orifices are present, the principal alternative diagnosis is the classical urogenital sinus, where the opening is located more anteriorly than in the posterior cloaca variant, typically in the position of a normal urethra, and which may be associated with other disorders of sexual development. 1 2

Since clinical examination generally allows reliable distinction of posterior cloaca from both penile agenesis and urogenital sinus, and assessment of the internal anatomy, including the gonads and Müllerian structures, can be performed at the time of diversion, we do not consider karyotype analysis mandatory in patients with posterior cloaca.

In keeping with the literature, hydrocolpos was found in all three cases with two of them also exhibiting additional Müllerian anomalies. Of interest, none of the three cases had significant structural cardiac and renal anomalies, and although renal dysfunction was a critical factor in the fatal outcomes, it occurred in the absence of major anatomical urological abnormalities. This observation supports the notion that renal insufficiency can result from functional rather than structural pathology, as previously described.

The terminology used to describe these variants is variable, contributing to uncertainty around the true incidence of posterior cloaca. Other terms used to describe the same defect include partial urorectal septal defect. 1 Cloacal malformations are considered by some to form part of the urorectal septum malformation sequence (URMS). 5 There are a few unique features common to our three cases that are in support of the association with the URMS, namely, the genital fold anomaly which lack corporal structures (effectively aphallia or absent penis), 1 the anteriorly displaced anal canal, and echogenic kidneys. The renal findings effectively demonstrate that there can be renal insufficiency without an anatomical urological abnormality. 2 5 Additional findings supportive of the URMS spectrum found in Case 2 include deficient abdominal wall musculature, sacral anomaly, and limb abnormalities; and in Case 3 include a single umbilical artery, solitary kidney, duplicated Y-shaped urethra, and pubic bone thickening. 5

Although the pathogenesis of the posterior cloaca remains debated, its clinical implication is clear. Management requires prenatal counseling where possible, comprehensive diagnostic evaluation, timely organ support, and a staged surgical approach. 6 The final goals of treatment in posterior cloaca are the same as other cloacal malformations: preservation of renal function, achievement of social continence, and satisfactory genital reconstruction. 6 7

Fecal stream diversion is usually described as a divided sigmoid colostomy; however, end-colostomy in the case of an adequate size perineal fistula is acceptable. 8 Urinary tract decompression may be facilitated by vaginostomy alone or may require an additional vesicostomy. 7 In our series, a large, atonic bladder that was difficult to catheterize was a common finding, and vesicostomy proved useful in facilitating bladder drainage and potentially preserving already compromised renal function. When assessing the Müllerian structures at initial surgery, vaginal septi must be taken down to adequately drain hydrocolpos in the case of hemivaginas. 1

Later in childhood, definitive reconstruction is guided by detailed imaging and endoscopy. Panendoscopy with catheter placement facilitates a cloacagram, and surgeon's presence during two-dimensional fluoroscopy can improve interpretation of overlapping structures. 8 However, even with endoscopy and cloacagram, accurately identifying and delineating urethral anomalies can be difficult despite strong clinical suspicion, as seen in our experience.

Reconstruction in classic posterior cloaca may be achieved with a sagittal trans-anorectal approach. 2 In posterior cloaca variants, where the anus is normally positioned within a functioning sphincter complex, it should be preserved to avoid muscle and nerve injury, 9 with only a total urogenital mobilization (TUM) being performed. 1 This allows the blood supply to the urethra and vagina to be left intact. 6 A rudimentary dorsal atretic urethra is a common finding in posterior cloacas, and it may be resected in the case of a good caliber functioning orthotopic urethra. 1 6 The thick pubic bone and cartilage hypertrophy may require carving to allow space for reconstruction. 9 External genital reconstruction depends on the available skin and presence of corporal structures or accessory urethrae. A feminizing genitoplasty has been described by splitting the end of the urogenital sinus to form labia minora. 6 In our third case, we describe a novel approach for genital reconstruction combined with the TUM. Using the fused labioscrotal folds we created labia minora around the vaginal introitus. This technique also allowed to achieve a visible urethral meatus, while preserving urethral length, which can be used to perform clean intermittent catheterization. 1 However, normal continence of stool and urine is expected in these patients, except in the case of sacral dysplasia. 6 In survivors, medium-term follow-up should focus on continence outcomes, while long-term care must consider menstrual and sexual health. 6

In conclusion, posterior cloaca variants are rare and complex anorectal malformations often associated with renal insufficiency. The phenotype described here, characterized by a midline labioscrotal sac, appears linked to poor renal outcomes. In survivors, individualized surgical planning, including total urogenital mobilization and reconstruction using the labioscrotal folds, can yield promising cosmetic results. This series expands the known spectrum of posterior cloaca and introduces a novel approach to genitoplasty. Long-term functional outcomes remain to be evaluated.
